# A Perspective of the Comprehensive and Objective Assessment of Analytical Methods Including the Greenness and Functionality Criteria: Application to the Determination of Zinc in Aqueous Samples

**DOI:** 10.3389/fchem.2021.753399

**Published:** 2021-10-14

**Authors:** Paweł Mateusz Nowak, Renata Wietecha-Posłuszny, Michał Woźniakiewicz, Aneta Woźniakiewicz, Małgorzata Król, Joanna Kozak, Marcin Wieczorek, Paweł Knihnicki, Justyna Paluch, Anna Telk, Karolina Mermer, Jolanta Kochana, Paweł Kościelniak, Janusz Pawliszyn

**Affiliations:** ^1^ Department of Analytical Chemistry, Faculty of Chemistry, Jagiellonian University, Kraków, Poland; ^2^ Department of Chemistry, University of Waterloo, Waterloo, ON, Canada

**Keywords:** green analytical chemistry, method assessment, RGB model, sustainable chemistry, validation, white analytical chemistry

## Abstract

The recently proposed concept of White Analytical Chemistry (WAC), referring to the Red-Green-Blue color model, combines ecological aspects (green) with functionality (red and blue criteria), presenting the complete method as “white”. However, it is not easy to carry out an overall quantitative evaluation of the analytical method in line with the WAC idea in an objective manner. This paper outlines the perspective of the future development of such a possibility by attempting to answer selected questions about the evaluation process. Based on the study consisting in the evaluation of selected model methods by a group of 12 independent analysts, it was shown how well individual criteria are assessed, whether the variability of assessments by different people is comparable for each criterion, how large it is, and whether averaging the scores from different researchers can help to choose the best method more objectively.

## Introduction

The overall evaluation of an analytical method according to strictly defined criteria is crucial in the process of comparing the available analytical procedures and in selecting the best one for a given application. Apart from the analytical criteria assessed during method validation determining the quality of the obtained results, it is necessary to consider other parameters influencing the method’s functionality. These parameters include cost, time, sample consumption, and other practical requirements, among others. Moreover, the overall assessment also requires considering the “green” criteria relating to the method’s environmental friendliness and safety (Koel and Kaljurand, 2006; Armenta et al., 2008; Gałuszka et al., 2013). Carrying out such an assessment in a comprehensive and reliable manner, without favoring any assessment criteria, requires the use of an appropriate tool and the adoption of certain unchanging rules (Nowak et al., 2020). As it is worth noting, the known greenness assessment scales allow the methods to be compared in terms of ecological aspects (Gałuszka et al., 2012; Tobiszewski, 2016; Płotka-Wasylka, 2018; Pena-Pereira et al., 2020), but they do not include analytical and practical parameters determining the functionality of the method.

One of the attempts to solve the above problem is the recently proposed concept of “White Analytical Chemistry” (WAC) (Nowak et al., 2021), which is associated with the long-known concept of “Green Analytical Chemistry” (GAC). WAC, like GAC, proposes a set of 12 invariant rules against which the method is evaluated. While in the case of GAC all refer to the ecological and safety aspects, WAC distinguishes only four most important and independent green rules, which are joined by 4 “red” rules - referring to analytical aspects, and 4 “blue” rules - referring to the practical aspects. It is a direct reference to the Red-Green-Blue color model (Nowak and Kościelniak, 2019), because the simultaneous fulfillment of the red, green and blue rules give the white color which means meeting all requirements and completeness, just like mixing light of these colors gives the impression of whiteness. The concept of WAC along with a dedicated RGB 12 algorithm encoded in an Excel spreadsheet available for everyone was discussed in detail in Nowak et al. (2021). Since this concept was introduced very recently, it has not yet been possible to develop unchangeable rules for assessing individual criteria, especially considering the huge variety of available methodologies. The process of awarding points for given criteria is therefore at this time more or less subjective. An attempt to develop such standards, aimed at increasing the objectivity of evaluation, is therefore very important task planned for the nearest future.

This paper attempts to address this challenge by answering selected questions relating to the method evaluation process in accordance with the WAC idea. It presents and discusses the results of the evaluation of eight selected model methods using the RGB 12 algorithm, which was performed independently by 12 analysts representing the same research unit (Department of Analytical Chemistry of the Jagiellonian University in Krakow), but different levels of experience in individual techniques. The main goal was to check, on the basis of all assessments, how well individual criteria are assessed - which ones are the best and which ones are the worst, and how the green criteria fall out against red and blue ones. Furthermore, it was aimed to find out what is the consistency of assessments of individual criteria by different people with different optics for given analytical techniques, and whether the involvement of a wide range of people in the evaluation can increase the objectivity of comparing methods and choosing the best overall. The aim of this study was not, however, a detailed analysis and comparison of individual methods [this was the purpose of another contribution (Kościelniak et al., 2021)], but focusing on the evaluation process itself.

## Methodology

The subject of the assessment were arbitrarily selected methods for determining zinc in water: spectrophotometric with diode array detection (DAD), fluorimetric, differential pulse voltametric, stripping potentiometric, flame atomic absorption spectrometric (FAAS), inductively coupled plasma optical emission spectrometric (ICP-OES), inductively coupled plasma mas spectrometric (ICP-MS) and electrophoretic. These methods included procedures used routinely at the Department of Analytical Chemistry of the Jagiellonian University, as well as published in scientific literature (Compañó et al., 1996; Azubel et al., 1999; Tarley et al., 2009; Lagerström et al., 2013; Lu et al., 2017; Paluch et al., 2020) Some of them were realized in the flow mode (fluorimetric, DAD, ICP-MS and electrophoretic).

The assessment procedure consisted in assigning scores to all eight methods, independently by all 12 people, for each of the 12 WAC rules (Nowak et al., 2021): R1 - scope of application (including the range of linearity, the number of analytes simultaneously determined, the range of tolerable composition of the sample matrix, selectivity, robustness), R2 - limit of detection and quantification (LOD and LOQ), R3 - precision, R4 - accuracy, G1 - reagent toxicity measured by the number of pictograms, G2 - amount of reagents and waste produced, G3 - consumption of energy and other media, G4 - direct impact on the user (safety), B1 - cost consumption, B2 - time consumption, B3 - requirements: sample consumption for analysis and other practical requirements of the method (assessed as two separate parameters) and B4 - operational simplicity: miniaturization, procedure integration/automation and instrument portability (assessed as three separate parameters). The awarded scores were on a scale of 0–120, where 0 is the worst possible result, 100 is a completely satisfactory result in the context of the planned application, and scores above 100 were awarded in special cases to emphasize the unique advantages of a given method. More details on the evaluation algorithm are available in Nowak et al. (2021). The parameters such as LOD and LOQ (R2), precision (R3), accuracy (R4), toxicity (G1), amount of reagents and waste (G2), occupational hazards (G4), cost (B1), time consumption (B2) and consumption of sample (B3) were pre-quantified from the literature data or estimates made for the evaluation, so that each evaluator would use the same values for these parameters. The remaining parameters were more qualitative than quantitative, therefore they were assessed intuitively.

In each case the number of awarded points was the individual decision of evaluator, not consulted with other participants. Such a subjective approach was intentional to make diagnose of the possible discrepancies coming from the assumed freedom. The abovementioned guideline that 100 means full appropriateness was the sole requirement.

## Results

**FIGURE 1:**
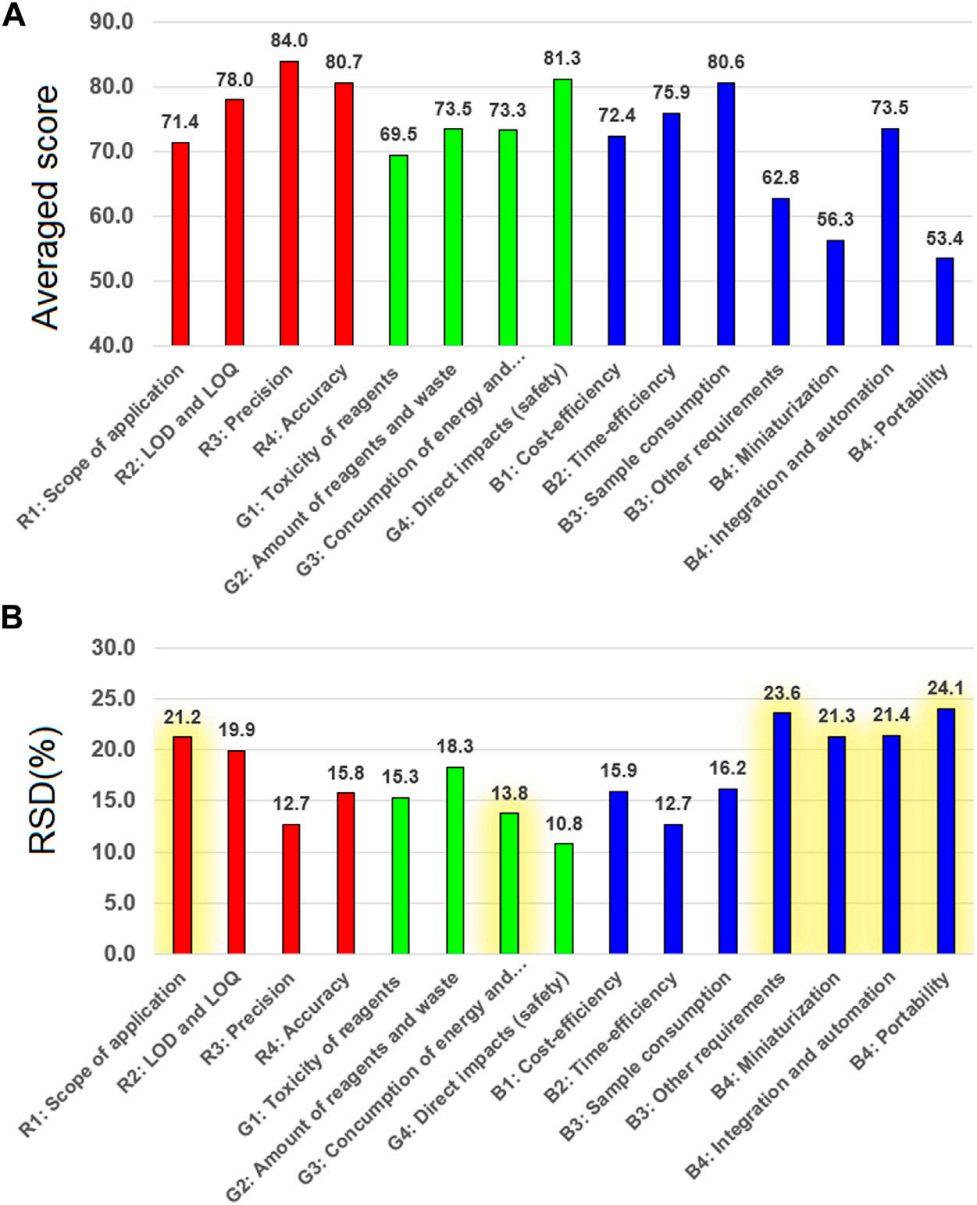
The average evaluation results of the eight model methods for the determination of zinc in water by 12 evaluators: **(A)** the average evaluation values for the individual parameters of the methods; **(B)** RSD(%) for the individual parameters resulting from the variety of scores awarded (*n* = 12). The qualitative parameters assessed intuitively are highlighted in yellow.

The results of the method evaluation are presented in Figure 1. Only those values are presented which, in the opinion of the authors, are crucial for the subject of this work. The additional data pertaining to the selected methods are shown in the Supporting Information (word file). A complete set of points awarded by each analyst is shown in the attached Excel file. Figure 1A shows the averaged values based on all methods and all evaluators, obtained for the particular criteria. In other words, it shows how well the individual parameters were assessed overall. Figure 1B shows the discrepancy of ratings for individual criteria as a relative percentage SD (RSD, for *n* = 12 meaning the number of evaluators), averaged for all assessed methods. Figure 1B shows the diversity of assessments resulting from the involvement of people with different experience and feelings about specific methods.

It can be observed that the green criteria were rated very similar to the red (analytical) criteria, in the range of about 70–85 points. This is a surprise, as it turns out that the available methods are, in the opinion of evaluators, as green as they are analytically effective. It is worth emphasizing that concern for ecological aspects is a relatively new trend in analytical chemistry, which could suggest shortcomings in this respect in the contemporary arsenal of available methods. Moreover, the assessed methods included procedures developed several or even over 10 years ago (Compañó et al., 1996; Azubel et al., 1999; Tarley et al., 2009). The best red and green criteria (above 80) were: precision, accuracy and occupational hazards, while the worst rated were the scope of application and the toxicity of the reagents. In the case of blue criteria, attention is drawn to the three parameters that are clearly rated the worst among all the colors: practical requirements (skills, facilities, equipment, infrastructure), miniaturization and portability. The consumption of the sample for analysis was assessed well, while the cost and time consumption were moderately satisfactory.

These results indicate, firstly, those features of the available methodologies which, in the context of expectations expressed in the degree of criticism of the assessment, are the worst. These are blue criteria relating to the generally understood simplicity of the method, requirements that we should meet as analysts and which our laboratory should meet, friendliness in terms of miniaturization and the possibility of transferring measuring equipment and conducting on-site measurements. Therefore, it can be presumed that, according to the average opinion of 12 evaluators, providing higher level of simplicity of analytical procedures and instrumentation may be the greatest challenge for the future development of available analytical techniques.

On the other hand, precision, accuracy, safety of use, sample consumption for analysis - these are the parameters that we judge best, and therefore perhaps should be treated with less priority in the context of further improvement, and our efforts should focus on other parameters. For example, scope of application, toxicity of reagents, waste production, time and cost of analysis, these may be other directions for further development, provided that good performance in terms of the abovementioned parameters is maintained. One can conclude that perhaps our attention is too much focused on “hard” validation criteria, which is probably dictated by various norms, standards and requirements set by scientific world. On that account, extending the validation process to include practical and ecological criteria could contribute to a more comprehensive and reliable view of the quality of the analytical method.

As for the consistency of assessments of individual criteria (Figure 1B), the largest average discrepancies were recorded for: scope of application, other practical requirements, miniaturization, integration/automation and portability. It is worth emphasizing that all the above-mentioned criteria were assessed qualitatively and intuitively, based on the knowledge and experience of the evaluators, which, as we know, could be different. These criteria were not quantified like the others. Criteria that were based on rigid values obtained during validation (LOD, precision, accuracy) were assessed in a more consistent manner, similarly as those based on the estimation results available to all evaluators (toxicity, waste, energy, cost, time, sample consumption). However, the differences measured by the average RSD values are not very large, for example, the difference between the criterion assessed most consistently (precision and time-efficiency) and the least consistent (portability) is 12.7 vs. 24.1%, although portability can be understood and treated very differently by individuals (the exact meaning of the criteria was not consulted during the assessment).

These data indicate a general need to limit the variability of the scores awarded to eliminate the dependence of the assessment on the subjective beliefs of the evaluator as much as possible. As can be seen, the criteria based on hard numbers and estimates were assessed more consistently, but still the RSD values clearly above 10% indicate the need to deal with this problem. The best solution seems to be to establish rigid evaluation guidelines indicating which scores should be awarded for given values of the numerical parameters assessed. For more qualitative criteria, such as portability and degree of miniaturization, the development of such guidelines would probably be much more difficult. A potential solution could be the prior assignment of given analytical techniques to given categories, so that the field for manipulation during their evaluation would be much smaller. For example, the ICP-MS method requiring advanced and large-sized instruments could be assigned to the category of “particularly not very portable methods,” for which the ratings could not be higher than a certain fixed value.

However, it is worth recognizing how difficult and responsible the task would be to develop a rigid framework for assessing individual criteria, considering the variety of techniques, analytical problems, sample matrices, etc. It seems, however, that the effort is worthwhile bearing in mind the advantage that it would undoubtedly be to conduct a fully objective evaluation of methods in a global manner, going beyond the validation criteria, greenness assessment scales, combining separate attributes that determine the quality of the method as a whole.

Another issue is the attempt to answer the question of whether the involvement of a large group of people to evaluate the same methods in a subjective manner can allow for increasing the reliability and objectivity of the assessment by basing on average ratings. Table 1 shows the normalized overall scores for the individual methods for the individual evaluators. These values are the arithmetic mean of the evaluation of all criteria, and according to the WAC concept, they are an indicator of the method’s “whiteness”, i.e., the degree of its completeness and balance. The normalization process consisted in the fact that the method best assessed by a given analyst was given a value of 100, and the results of the other methods were proportionally recalculated.

**TABLE 1:** The overall assessment values for the individual methods according to all evaluating people, including mean values, RSD, and the resulting position in the ranking.

Method	Fluorimetric	DAD	Voltametric	Potentiometric	FAAS	ICP-OES	ICP-MS	Electrophoretic
Analyst 1	100.0	97.6	96.3	97.5	97.5^a^	96.4^a^	88.2^a^	88.6^a^
Analyst 2	97.1	100.0	94.2	90.9	87.3	85.2	69.5	82.9
Analyst 3	100.0	97.5	99.0	93.7	89.4	89.2	78.9	82.5
Analyst 4	100.0	96.1	92.4	90.5	89.1	85.1	80.6	83.6
Analyst 5	100.0	86.8	99.4	76.4^a^	70.8^a^	71.7^a^	74.9	52.0^a^
Analyst 6	100.0	97.9	95.3	87.6	76.2^a^	80.0	64.9	57.2^a^
Analyst 7	93.6	90.5	100.0	94.2	88.3	78.3	67.9	73.2
Analyst 8	100.0	91.1	84.0^a^	84.0	97.6^a^	83.5	59.4^a^	66.4
Analyst 9	100.0	94.8	98.1	96.1	91.2	91.2	82.3	83.4
Analyst 10	99.8	100.0	97.2	94.4	91.2	88.7	83.5	89.7^a^
Analyst 11	100.0	96.6	94.8	83.6	86.5	79.8	77.6	69.6
Analyst 12	100.0	87.9	81.3^a^	66.8^a^	82.0	73.9	70.6	54.1^a^
Mean	99.2	94.7	94.3	88.0	87.3	83.6	74.9	73.6
RSD(%)	2.0	4.8	6.3	10.3	9.0	8.7	11.4	18.5
Position	I	II/III	II/III	IV/V	IV/V	VI	VII/VIII	VII/VIII

a Outliers indicated arbitrarily as the values different of more than 10 from the mean.

As can be seen from Table 1, despite some differences in the assessment of individual methods by different people, the general trend that classifies the methods from the best to the worst is clearly visible: Fluorimetric > DAD ex aequo with Voltametric > Potentiometric ex aequo with FAAS > ICP-OES > ICP-MS ex aqeuo with Electrophoretic. This is clearly visible from the mean values obtained by individual methods together with the RSD values. This analysis makes it possible to select the best overall method in an unambiguous and highly objective manner - it is the Fluorimetric method, with an average overall score close to 100 and a very low RSD value. Table 1 also shows the outliers - results that differ from the mean value for a given method by more than 10, which, as it occurs, are grouped for certain evaluators and methods (the most outliers were recorded for Analyst five and Electrophoretic method). This suggests that some people may have quite different opinions about some methods, which is obvious when one considers the large group of people involved and their various experiences. Analysts working with specific techniques tend probably to favor them. The likely reason is that on the one hand, they know the shortcomings of their techniques very well, but on the other hand, they know how to minimize them, what to do to make the method better. Operators with no experience have only general theoretical knowledge and basic practical skills, so they may assess the method less favorably. However, there were relatively few results that are significantly different from the rest. In such a situation, relying on average ratings seems to be a good idea, maybe not ideal, but it significantly increases the level of objectivity of the assessment. Obviously, averaging makes more sense the larger the number of evaluators is. In everyday life, it may not always be possible and convenient to conduct an assessment independently by many people. We suggest at least three independent evaluators. It seems to be a good temporary solution until objective assessment guidelines will be developed, especially in exceptional situations, e.g., comparison of a new method with known alternatives in the publication to prove its superiority. Noticeably, the scientific world tends to use averaging very widely as the best mathematical method to cope with variability of results. It is also worth noting that in this particular case the evaluators were not required to have extensive experience in working with each technique, it was a test showing the general idea. In practice, the quality of the assessment process by many people can be improved by involving specialists in all the assessed methods.

## Summary

The evaluation of analytical methods according to the WAC concept, based on the 12 general principles divided into the three separate categories marked by colors, gives the possibility of a holistic view, combining green and functional (red and blue) aspects. Until rigid standards for evaluating individual criteria will be developed, the process is subjective, so the results of the evaluations should be treated with an appropriate detachment. The idea of WAC, however, necessitates to refer to all relevant parameters fairly, without favoring any category. It can also be treated as an extension of the validation process with other criteria that have not been considered so far in the publications on new methods, e.g., cost-effectiveness, time-consumption, amount of waste, operational simplicity, etc. Undoubtedly, one should take more care of these sometimes overlooked criteria, which may contribute to increasing the overall quality and attractiveness of the developed analytical methods. Efforts to increase the objectivity of evaluation are, however, necessary and are the nearest perspective for the development of the WAC concept. However, this should not discourage publishing the results of assessments carried out in a subjective manner, as only on the basis of them will it be possible to develop these standards in the future. Importantly, one should simultaneously publish the data based on of which specific scores have been assigned. The readers interested in given methodologies should have an insight into the estimates of analysis costs, analysis time, toxicity, waste, etc., and the estimation process should be clearly described and carried out with due care in scientific publications.

Increasing the objectivity of the assessment can also be achieved by involving an extended group of analysts who evaluate selected methods independently, based, however, on the same values of quantitative parameters. Averaging the ratings may allow for a more reliable selection of the optimal method, and also to indicate the most outliers in terms of quality. The level of objectivity of such an assessment can be expressed, for example, by the RSD of the ratings given by different people. In a situation such as that described above - where the Fluorimetric method obtained a score of 99.2/100 with the RSD 2.0 value, it may allow for an unambiguous and convincing indication of the best method globally - green and functional, i.e., white. Nevertheless, besides overall assessment expressed by whiteness, one should always ensure that the key performance criteria, which may pose a bottleneck for using the method in practice, are at least acceptable. The assessment results should be treated as a valuable support and not an absolute obligation to choose a specific method, it is important to use common sense and refer to reality.

Finally, it should be emphasized that obtaining a good concordance of ratings will always be easier when we confront many methods with each other, and more difficult in the case of a single method assessment. Therefore, it seems a good idea to choose a “gold method”, which is standardly used in a given case, well known, and which is easy to assess by a wide range of researchers. Then the evaluation of the new method may be easier by its direct reference to such reference method. More detailed advice on how to use the RGB 12 model to evaluate methods according to the WAC concept will be proposed by us in the near future.

## Data Availability

The raw data supporting the conclusion of this article will be made available by the authors, without undue reservation.
