# Supplementary material for: A Perspective of the Comprehensive and Objective Assessment of Analytical Methods Including the Greenness and Functionality Criteria: Application to the Determination of Zinc in Aqueous Samples
Source: Front Chem. 2021 Oct 14;9:753399. doi: 10.3389/fchem.2021.753399 (PMC8551957; doi:10.3389/fchem.2021.753399)
Supplement: Supplementary file 1 [file DataSheet1.docx]

Supplementary material

A Perspective of the Comprehensive and Objective Assessment of Analytical Methods Including the Greenness and Functionality Criteria: Application to the Determination of Zinc in Aqueous Samples

Paweł Mateusz Nowak^1^, Renata Wietecha-Posłuszny^1*^, Michał Woźniakiewicz^1^, Aneta Woźniakiewicz^1^, Małgorzata Król^1^, Joanna Kozak^1^, Marcin Wieczorek^1^, Paweł Knihnicki^1^, Justyna Paluch^1^, Anna Telk^1^, Karolina Mermer^1^, Jolanta Kochana^1^, Paweł Kościelniak^1^, Janusz Pawliszyn^2^

^1^Department of Analytical Chemistry, Faculty of Chemistry, Jagiellonian University in Kraków, 2 Gronostajowa St., 30-387, Kraków, Poland

^2^Department of Chemistry, University of Waterloo, 200 University Avenue West, Ontario, N2L 3G1, Canada

*** Correspondence:**Renata Wietecha-Posłuszny

renata.wietecha-posluszny@uj.edu.pl

**Table S1.** The analytical methods evaluated during the study.

| **Abbreviation,**  **measurement system** | **Sample** | **Procedure** | **Ref.** |
| --- | --- | --- | --- |
| SIA-CE/DAD,  UV/VIS-DAD | Drinking and waste water | Flow module on line connected with a vaporizer to concentrate the sample and off-line with capillary electrophoresis to separate the sample components | [1] |
| FAAS, AAS | Waters | The ISO procedure experimentally verified by authors in terms of quantitative parameters using PinAAcle 900 spectrometer (Perkin Elmer Inc., USA) | [2] |
| ICP/OES, OES | Waters | The ISO procedure experimentally verified by authors in terms of quantitative parameters using Optima 2100 DV spectrometer (Perkin Elmer, Inc, USA) | [3] |
| SP, potentiometry | Lake and effluent water | Procedure based on simultaneous preconcentration and reduction of metal ions onto a multiwall carbon nanotube electrode followed by subsequent chemical stripping | [4] |
| DPASV,  voltammetry | Lake water | Procedure based on the use of a disposable sensor—a screen-printed carbon electrode co-modified with an in situ plated bismuth film and gold nanoparticles | [5] |
| FIA-DAD,  UV/VIS -DAD | River water | Procedure based on the use of PAR (4-(2-pyridylazo) resorcinol) as colorimetric reagent and multivariate calibration for the determination of Zn, Cu and Mn in river water samples | [6] |
| FIA-SF,  spectrofluorimetry | Food | Procedure based on the fluorescence of the zinc-8-(benzenesulphonamido) quinoline chelate in a micellar medium of sodium dodecylsulfate | [7] |
| FIA-ICP/MS, MS | Ocean seawater | Preconcentration of metals using a column with chelating resin | [8] |

SIA-CE/DAD—sequential injection analysis-capillary electrophoresis with diode array detection; FAAS—flame atomic absorption spectrometry; ICP/OES—inductively coupled plasma/optical emission spectrometry; SP—stripping potentiometry; DPASV—differential pulse anodic stripping voltammetry; FIA-DAD—flow injection analysis with diode array detection; FIA-SF—flow injection analysis with spectrofluorimetric detection; FIA-ICP/MS—flow injection analysis-inductively coupled plasma mass spectrometry.

**Table S2.** Values of the key parameters characterizing the particular methods.

| **Method** | **LOD (µg/L)** | **RSD (%)** | | **Relative Error (%)** | | **Total Number of Pictograms** | **Waste Production (mL/10 Samples)** | **Occupational Hazards** | | **Estimated Cost (EUR)** | **Estimated Speed of Analysis (s Per Sample)** |
| --- | --- | --- | --- | --- | --- | --- | --- | --- | --- | --- | --- |
| SIA-CE/DAD | 25 | | 6.0 | | 7.8 | 14 | 190 | | 3 | 20 | 60 |
| FAAS | 30 | | 2.8 | | 3.0 | 4 | 225 | | 4 | 50 | 1 |
| ICP/OES | 3 | | 3.0 | | 5.0 | 4 | 230 | | 4 | 250 | 2.5 |
| SP | 28 | | 5.6 | | 2.1 | 8 | 300 | | 1 | 12 | 11 |
| DPASV | 0.05 | | 2.8 | | 15.0 | 6 | 300 | | 1 | 12 | 7.5 |
| FIA-DAD | 72 | | 3.7 | | 12.0 | 4 | 110 | | 0 | 9 | 1 |
| FIA-SF | 0.2 | | 1.1 | | 0.6 | 14 | 110 | | 0 | 11 | 1.3 |
| FIA-ICP/MS | 0.001 | | 3.0 | | 1.0 | 19 | 900 | | 3 | 490 | 8.8 |

Data taken from *Kościelniak, P.; Nowak, P.M.; Kozak, J.; Wieczorek, M. Comprehensive Assessment of Flow and Other Analytical Methods Dedicated to the Determination of Zinc in Water. Molecules 2021, 26, 3914. https://doi.org/10.3390/molecules26133914*

References

1. Paluch, J.; Kozak, J.; Wieczorek, M.; Woźniakiewicz, M.; Gołąb, M.; Półtorak, E.; Kalinowski, S.; Kościelniak, P. Novel ap-proach to sample preconcentration by solvent evaporation in flow analysis. Molecules 2020, 25, 1886.

2. Water Quality—Determination of Cobalt, Nickel, Copper, Zinc, Cadmium and Lead—Flame Atomic Absorption Spectro-metric Methods; PN-ISO 8288:2002. Available online: https://www.iso.org/standard/15408.html (accessed on).

3. Water Quality—Determination of Selected Elements by Inductively Coupled Plasma Optical Emission Spectrometry (ICP-OES); PN-EN ISO 11885:2009. Available online: https://www.iso.org/standard/36250.html (accessed on).

4. Azubel, M.; Fernández, F.M.; Tudino, M.B.; Troccoli, O.E. Novel application and comparison of multivariate calibration for the simultaneous determination of Cu, Zn and Mn at trace levels using flow injection diode array spectrophotometry. Anal. Chim. Acta 1999, 398, 93–102.

5. Compañó, R.; Ferrer, R.; Guiteras, J.; Prat, M.D. Flow injection method for the fluorimetric determination of Zn with 8-(Benzenesulphonamido) Quinoline. Microchim. Acta 1996, 124, 73–79.

6. Lagerström, M.E.; Field, M.P.; Séguret, M.; Fischer, L.; Hann, S.; Sherrell, R.M. Automated on-line flow-injection ICP-MS determination of trace metals (Mn, Fe, Co, Ni, Cu and Zn) in open ocean seawater: Application to the GEOTRACES program. Mar. Chem. 2013, 155, 71–80.

7. Tarley, C.R.T.; Santos, V.S.; Baêta, B.E.L.; Pereira, A.C.; Kubota, L.T. Simultaneous determination of zinc, cadmium and lead in environmental water samples by potentiometric stripping analysis (PSA) using multiwalled carbon nanotube electrode. J. Hazard. Mater. 2009, 169, 256–262.

8. Lu, Z.; Zhang, J.; Dai, W.; Lin, X.; Ye, J.; Ye, J. A screen-printed carbon electrode modified with a bismuth film and gold nanoparticles for simultaneous stripping voltammetric determination of Zn(II), Pb(II) and Cu(II). Microchim. Acta 2017, 184, 4731–4740.
